# Effect of a Composite Activator on Comprehensive Performance of Alkali-Activated Foam Concrete

**DOI:** 10.3390/ma19081616

**Published:** 2026-04-17

**Authors:** Zhongshuai Hu, Yuanliang Xiong, Yuchen Cai, Shaoyuan Zheng, Yuting Lv, Yan Li, Xinrong Zhao, Yongkang Wang, Liguo Ma

**Affiliations:** 1 School of Energy and Constructional Engineering, Shandong Huayu University of Technology, Dezhou 253034, China17862686173@163.com (Y.C.); 15686258069@163.com (Y.L.); empress1232025@163.com (Y.L.); 18769617959@163.com (X.Z.); 19560659198@163.com (Y.W.); 2 School of Civil Engineering, Yantai University, Yantai 264005, China

**Keywords:** composite activator, stability, compressive strength, drying shrinkage

## Abstract

This study investigates the synergistic mechanism between composite activators (NaOH and Na_2_SiO_3_ blend) and the microstructure–macroperformance relationship of foam concrete, focusing on the influence of different activator dosages on foam concrete stability, compressive strength, and drying shrinkage behavior. Experimental results indicate that excessively high activator dosages impair foam concrete stability, reduce compressive strength, and accelerate drying shrinkage. However, an appropriate amount of composite activator effectively improves the stability of freshly mixed foam concrete, significantly reducing settlement rates. During the hardening stage, it optimizes pore size distribution, promotes the formation of denser hydration products, and enhances the mechanical properties of the pore framework, thereby synergistically improving the mechanical performance of foam concrete.

## 1. Introduction

Faced with the critical global challenge of the climate emergency, leading figures in the scientific community have issued an urgent appeal. Professor Pierre Humbert of the University of Oxford noted in 2019 that the climate crisis demands immediate and decisive action, describing the situation as one that urgently requires a wake-up call [1]. Concrete accounts for a significant proportion of global carbon dioxide emissions; therefore, the use of solid waste in its production is particularly crucial.

Alkali-activated foam concrete (AAFC) is a lightweight porous material prepared from solid wastes such as slag, fly ash, and metal scrap. Compared with traditional ordinary Portland cement-based foam concrete, AAFC offers advantages including short setting time and high early strength, demonstrating broad application prospects in the field of building materials [2,3]. Furthermore, AAFC exhibits characteristics such as low cost [4,5]. However, compared to Portland cement-based foam concrete, it still suffers from issues including poor stability, insufficient mechanical properties, and high drying shrinkage [6,7,8], which severely limit its further promotion and application.

The performance and applications of AAFC largely depend on the stability of freshly mixed foam concrete, its pore structure after hardening, and the gel structure of its matrix [9]. A major current challenge lies in the instability of the matrix yield stress. Simultaneously, the high ionic concentration and surface tension in the alkaline environment cause bubble instability, leading to bubble rupture, coalescence, and upward migration. This results in oversized or non-uniform pore structures in hardened foam concrete, severely compromising its mechanical properties, particularly compressive strength [10,11,12,13]. Furthermore, the dense molecular structure and high moisture evaporation rate of geopolymer gels often result in significant autogenous shrinkage and drying shrinkage [14,15], increasing the risk of cracking.

To address the aforementioned issues with AAFC, optimizing the activator formulation is particularly crucial. Existing research has largely focused on single activators or slurry and mortar systems; there is a lack of systematic understanding of the synergistic mechanisms of composite activators within the complex, multiphase system of foamed concrete. In particular, there is insufficient research into the intrinsic relationships between these activators and bubble stability, pore structure evolution, and drying shrinkage behavior, with most studies remaining at the level of qualitative descriptions of macroscopic properties. And traditional single activators often struggle to synergistically regulate the workability, reaction kinetics, and microstructural development of freshly mixed foam concrete. For instance, NaOH provides high alkalinity to promote dissolution but readily causes foam collapse and tends to form loose gel products resembling N-A-S-H; Na_2_SiO_3_ aids in forming high-strength cohesive products but exhibits insufficient dissolution capacity and high viscosity [16,17], In recent years, composite activators have garnered significant attention due to their ability to synergistically combine multiple alkaline components. Numerous scholars have conducted extensive research on alkali-activated foamed concrete. Dang et al. systematically investigated the effects of the modulus of the composite activator and the Na_2_O concentration on the properties and pore structure of fresh AAFC mixtures, finding that although increasing the alkali content shortens the setting time, it reduces foam stability and leads to an increase in pore size [18]. With regard to composite activators, researchers are exploring the synergistic effects of multi-component alkaline components and solid waste-based precursors; for example, utilizing NaOH and Na_2_SiO_3_ to activate a fly ash–slag–rice husk ash system to achieve synergistic optimization of strength and density under room-temperature curing [19]. The aforementioned studies confirm that the synergistic regulation of composite activators is key to optimizing the performance of AAFC; however, systematic research into the quantitative relationship between activator dosage and bubble stability, pore structure evolution, and macroscopic properties remains relatively scarce. A well-designed activator blend not only regulates the reaction process and enhances bubble stability but also promotes the formation of a denser, stronger reaction product matrix. However, excessive activator dosage can compromise the performance of foam concrete, necessitating precise control of its content.

To this end, this study utilized slag-silicate cement as a precursor to systematically investigate the role of composite activators at different NaOH dosages. It innovatively established a correlation between ‘activator dosage—pore structure characteristics—macroscopic properties’, revealing that an optimal dosage (3% NaOH) enhances performance by optimizing the uniformity of the pore structure and promoting the formation of highly polymerized C-(A)-S-H gel to achieve performance enhancement, thereby providing a theoretical basis for the refined design and performance regulation of composite activators in alkali-activated foamed concrete. These findings can directly guide the optimization of mix designs for both precast components and in situ casting projects. By ensuring lightweight, high-strength properties and low shrinkage, they enable the effective utilization of industrial solid waste such as slag, thereby reducing raw material costs and carbon emissions, and promoting the large-scale application of high-performance alkali-activated foamed concrete in green building.

## 2. Experimental Method

### 2.1. Materials

This study employed S95-grade blast furnace slag and P·O 42.5 Portland cement to prepare AAFC (the S95 slag was sourced from Qiangdong Mineral Products Processing Plant in Lingshou County, Shijiazhuang, China; the P·O 42.5 Portland cement was sourced from 97 Building Materials Co., Ltd. in Zhucheng, Weifang, China). The density of the slag was 2.82 g/cm^3^, while that of the cement was 3.11 g/cm^3^. The chemical compositions of the slag and cement are shown in Table 1. A liquid water glass with a modulus of 1.5 and a concentration of 30% (the water glass was sourced from Yourui Refractory Materials Co., Ltd. in Jiashan County, Jiaxing, China), along with a self-prepared sodium hydroxide solution at 33% concentration, were used as activators (the sodium hydroxide was sourced from the Shanghai Chemical Reagents Procurement and Supply Station of China National Pharmaceutical Group, Shanghai, China). Foam was prepared using a synthetic surfactant modified with nano-Al_2_O_3_ (the synthetic surfactant is a proprietary product developed by Southeast University in Nanjing, China).

### 2.2. Sample Preparation

The relationship between foam concrete and target density is shown in Table 2. The preparation process for foam concrete is as follows: First, a foam is prepared by dissolving nano-Al_2_O_3_-modified synthetic surfactant in water at a ratio of 1:300. Cement (OP) and ground granulated blast furnace slag (GBFS) are mixed for 30 s using a mixer (60 rpm) to produce a uniform mixture. This dry mixture is dissolved in water and stirred, with sodium hydroxide solution and sodium silicate solution added during mixing to form a homogeneous slurry. Finally, the foam is incorporated into the slurry to prepare fresh AAFC.

### 2.3. Test Methods

#### 2.3.1. Stability Testing of Matrix and Fresh Foam Concrete

**Figure 1:**
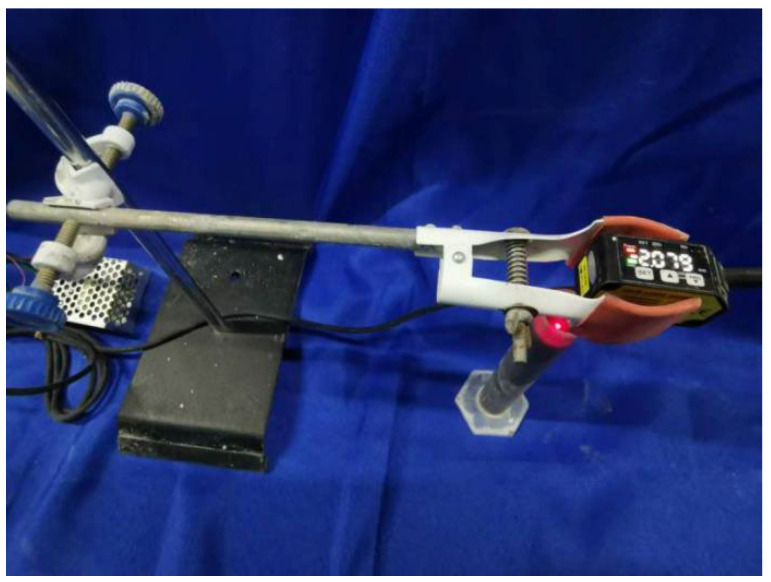
Experimental setup for foam concrete settlement.

Settlement was recorded using a laser displacement sensor (the laser displacement sensor is manufactured by Jingjiake Sensor Co., Ltd., based in Guangming District, Shenzhen, China; the model number is BX-LV30.) to evaluate the stability of the matrix and fresh foam concrete (as shown in Figure 1). Settlement data for both the matrix and fresh foam concrete were recorded at 5, 10, 20, 30, 60, and 90 min.

#### 2.3.2. Setting Time of Matrix

Testing performed using a Vicat apparatus in accordance with GB/T 1346-2011 [20] (Vicat apparatus is manufactured by Lanjian Zhongyi Instrument Equipment Co., Ltd. in Cangzhou, Hebei Province, China).

#### 2.3.3. Matrix Yield Stress Testing

The yield stress of the matrix was tested using the mini-cone test proposed by Roussel et al. [21,22]. A truncated cone mold (bottom diameter 100 mm) was employed for the test. Freshly mixed matrix was poured into the truncated cone mold until it was completely filled. The mold was then lifted vertically. After allowing the mortar to flow for 2 min, the spread diameter was measured at different time intervals. The yield stress was calculated using Equation (1), which relates the yield stress to the poured volume (*V*), density (*ρ*), gravitational acceleration (*g*), and spread radius (*R*).

(1) $$\tau =\frac{225\rho {\mathrm{gV}}^{2}}{128\pi^{2}R^{5}}$$

#### 2.3.4. XRD and TG Experiments on Foam Concrete

A Bruker D8 Discover X-ray diffractometer was used to test the phases in the samples (Bruker D8 Discover X-ray diffractometer is manufactured by Bruker in Mannheim, Germany, specifically by its subsidiary Bruker AXS GmbH, Billerica, MA, USA). XRD scans were performed between 5° and 90° at a step size of 0.02°. A TA Discovery TGA 550 thermogravimetric analyzer was used to record mass loss of the material (TA Discovery TGA 550 thermogravimetric analyser is manufactured by TA Instruments, a subsidiary of Waters Corporation in the Milford, MA, USA).

#### 2.3.5. Bubble Evolution, Microstructure, and Pore Structure of Foam Concrete

Bubble evolution in fresh HAAFC was examined using an optical microscope (Microscope (RY605) is manufactured by Shanghai Renyue Electronic Technology Co., Ltd. in Shanghai, China). The same optical microscope (RY605) was also employed to assess the pore size distribution of hardened HAAFC. Samples (30 mm × 30 mm diameter) cut from the center of a 30 mm × 300 mm specimen were used to test the pore size distribution of hardened HAAFC. The 30 mm × 30 mm sample was polished using 2000-grit fine sandpaper and then cleaned with acetone.

#### 2.3.6. Compressive Strength and Drying Shrinkage of Hardened Foam Concrete

The compressive strength of foam concrete was tested using 70.7 mm × 70.7 mm × 70.7 mm specimens at 7 days, 14 days, and 28 days. Prior to compressive strength testing, specimens were dried at 60 °C. Three specimens were used to calculate the average compressive strength.

Dry shrinkage of the foam concrete was determined using 40 mm × 40 mm × 160 mm specimens according to GB/T 11969-2008 [23]. The drying shrinkage of foam concrete at 3, 7, 14, 28, 35, 42, and 49 days was evaluated by measuring the length change in specimens cured at 20 ± 2 °C and 43 ± 2% humidity. Three specimens were used to calculate the average drying shrinkage.

#### 2.3.7. Experiment on the Water Absorption of Foam Concrete

The 3-day water absorption rate of foam concrete was tested at the 28-day age using specimens measuring 70.7 mm × 70.7 mm × 70.7 mm. Specimens were dried at 60 °C prior to testing. Three specimens were used to calculate the average water absorption rate of foam concrete. The specific procedure followed JGJ/T 341-2014 [24].

## 3. Results and Discussion

### 3.1. Effect of Activator on Matrix Stability

**Figure 2:**
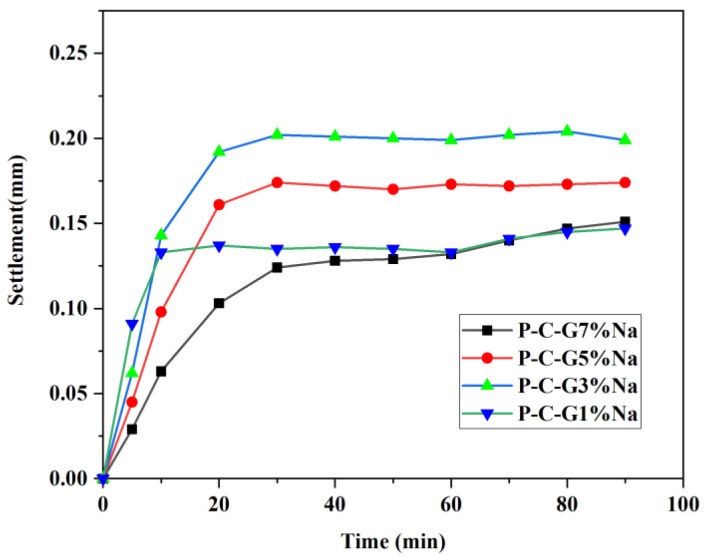
Settling of a foam-free matrix.

The settlement of the matrix at different activator dosages is shown in Figure 2. It can be observed that as the activator dosage increases, the settlement of the matrix first increases and then decreases. The chemical shrinkage of alkali-activated materials is significantly higher than the settlement of the matrix in Figure 2 [25,26]. Therefore, bleeding is the cause of settlement in the matrix [10].

### 3.2. Stability of Fresh Foam Concrete

**Figure 3:**
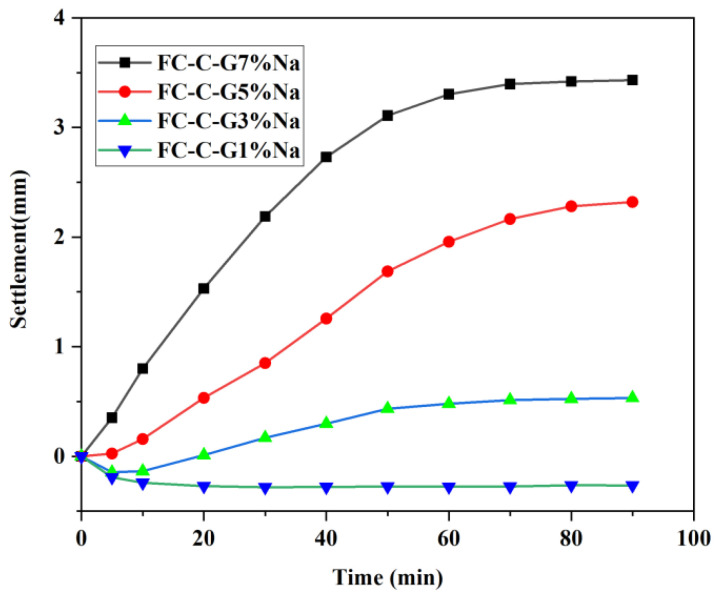
Settlement of freshly mixed foam concrete.

The settlement of fresh foam concrete is shown in Figure 3. The 90 min settlements for FC-C-G7%Na, FC-C-G5%Na, FC-C-G3%Na, and FC-C-G1%Na were 3.432 mm, 2.349 mm, 0.501 mm, and −0.250 mm, respectively. It was observed that as the activator dosage decreases, the settlement of freshly mixed foam concrete also diminishes. The FC-C-G1%Na group exhibited the smallest settlement and reached stability in the shortest time (approximately 15 min), indicating the highest stability. The destabilizing forces on bubbles can be counteracted by the formation of interstitial paste [27,28]. The yield stress of the matrix may account for this behavior, which will be elaborated in the next section.

### 3.3. Effect of Matrix Yield Stress Development on Foam Concrete Stability

The flowability and corresponding yield stress are shown in Table 3. Both flowability and yield stress vary with different matrix compositions. Reducing the activator dosage increases the flow rate evolution of the matrix, thereby accelerating the rise rate of the matrix yield stress. Meanwhile, the addition of sodium hydroxide reacts with the slurry to generate water [29], and the increase in water content can lead to enhanced slurry flowability [30], which may explain this phenomenon. Beyond the direct influence of the activator on the foam, the yield stress of the matrix may be responsible for this behavior, as discussed in the next section.

### 3.4. Pore Formation and Stability Mechanisms in Foam Concrete

Research addresses the collapse of freshly mixed foam concrete due to drainage. Foam expulsion is regarded as fluid flow within a porous medium. Flow is prohibited when the matrix yield stress (τ) exceeds the critical yield stress (τ_0_). The foam’s τ_0_ can be calculated using Equation (2) [10].

(2) $$\tau_{0}≅\rho_{1}gr$$

In the equation, τ_0_ denotes the critical yield stress of the foam, ρ_1_ represents the fluid density (ρ_1_), g is the gravitational acceleration, and r is the pore radius. Within the foam, r can be regarded as the maximum radius of pores through which fluid can flow in the porous medium. According to Equation (3) [31], r can be calculated using the bubble radius R and the foam volume fraction ϕ.

(3) $$r=R\frac{0.27\sqrt{1-\phi }+3.17{\left(1-\phi \right)}^{2.75}}{1+0.57{\left(1-\phi \right)}^{0.27}}$$

**Figure 4:**
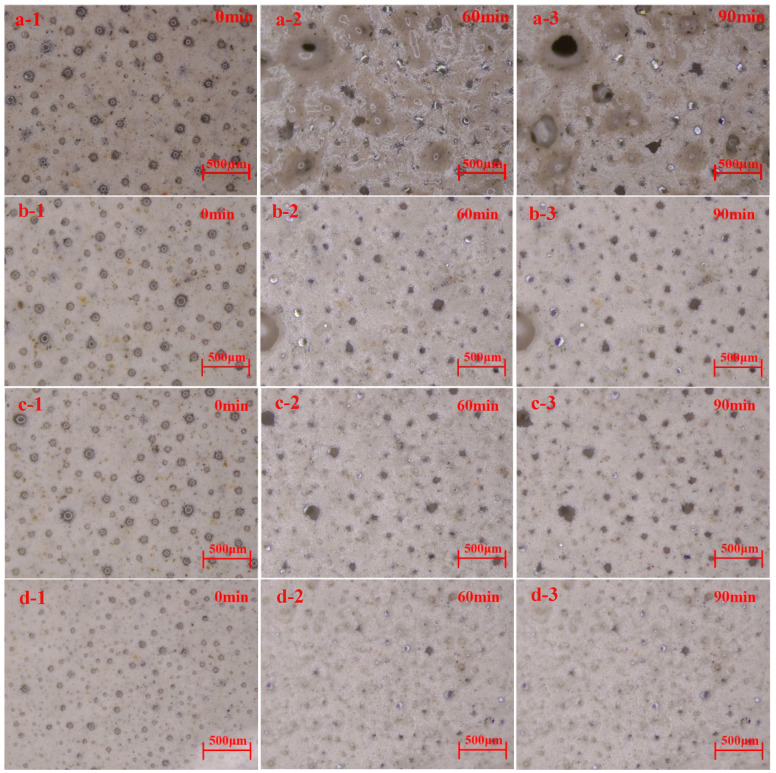
Evolution of bubbles in fresh foam concrete. (**a1**–**a3**) FC-C-G7%Na, (**b1**–**b3**) FC-C-G5%Na, (**c1**–**c3**) FC-C-G3%Na, (**d1**–**d3**) FC-C-G1%Na.

The evolution of bubbles at 0, 60, and 90 min is shown in Figure 4. As bubble diameter increases, the yield stress fluid becomes easier to drain. Therefore, the critical yield stress τ_0_ is calculated using the maximum bubble radius of 65 μm. Using R ≈ 68 μm, ϕ = 55%, and ρ_1_ = 1800 kg/m^3^ in Equations (2) and (3), r and τ_0_ can be obtained. τ_0_ is calculated as 0.44 Pa.

τ is significantly higher than τ_0_, indicating that foam drainage in fresh foam concrete can be suppressed. FC-C-G1Na exhibits the highest yield stress. As yield stress increases, the constraint force exerted by the matrix on bubbles may significantly rise. FC-C-G1Na, possessing the highest constraint force, tends to confer the greatest stability to FC-C-G1Na.

At the same magnification, the evolution processes of bubbles in different groups exhibit significant differences. In situ observation images at 90 min reveal that all bubble clusters gradually evolve into pores. Figure 4 clearly shows that reducing the activator dosage results in smaller pores formed at 90 min. This may be related to the higher matrix yield stress.

### 3.5. Statistics on the Pore Size of Foamed Concrete

**Figure 5:**
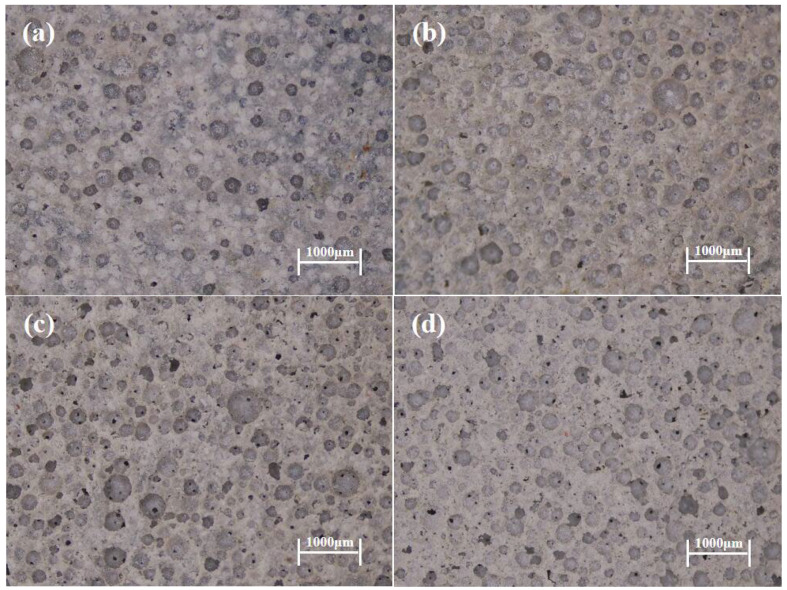
2D pore structure image of foam concrete. (**a**) FC-C-G7%Na, (**b**) FC-C-G5%Na, (**c**) FC-C-G3%Na, (**d**) FC-C-G1%Na.

**Figure 6:**
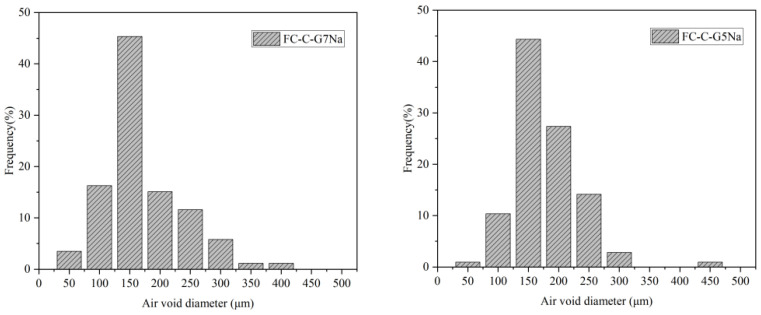

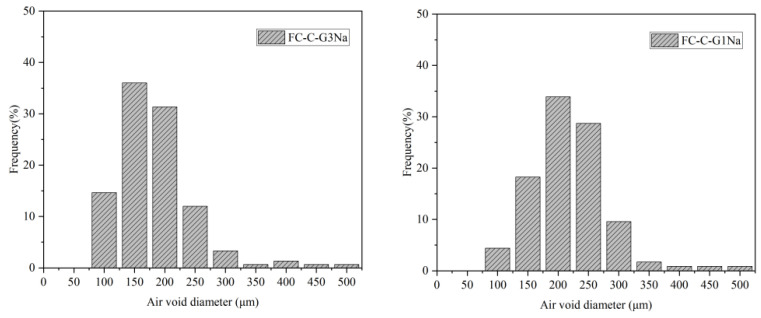
Pore size distribution of foam concrete.

Figure 5 shows 2D images of foam concrete pore structures at different foaming agent dosages. Pore counts were performed using Image Pro Plus software (version 6.3) 4KHDL. The pore size distributions for each group are presented in Figure 6. The proportions of pores with diameters ranging from 0 μm to 150 μm for FC-C-G7%Na, FC-C-G5%Na, FC-C-G3%Na, and FC-C-G1%Na were 65.12%, 55.66%, 50.67%, and 22.61%, respectively. As the sodium hydroxide dosage decreased, smaller pores were significantly reduced. This indicates that under composite activation conditions, a lower sodium hydroxide content tends to form a coarser pore structure.

The matrix setting times obtained from the Vicat apparatus are listed in Table 4. As shown in Table 4, increasing the activator dosage shortens the slurry setting time. Rapid setting effectively fixes bubbles before they fully coarsen, thereby inhibiting further development of interconnected pore channels and facilitating the formation of a complete pore structure in foam concrete [32]. This likely explains the observed pore structure characteristics.

### 3.6. Pore Wall of Foam Concrete

**Figure 7:**
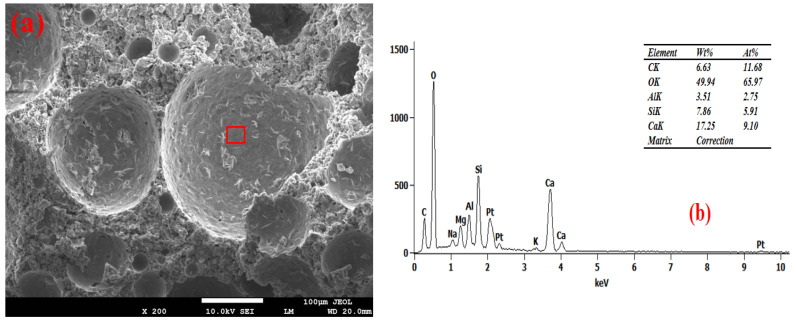
Microstructure of FC-C-G7Na. (**a**) SEM, (**b**) EDS spectrum.

**Figure 8:**
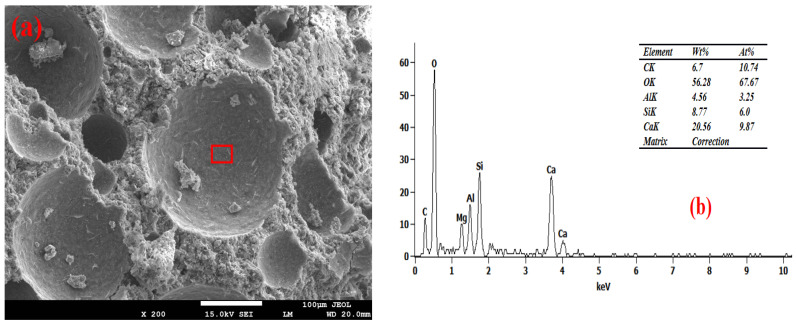
Microstructure of FC-C-G5Na. (**a**) SEM, (**b**) EDS spectrum.

**Figure 9:**
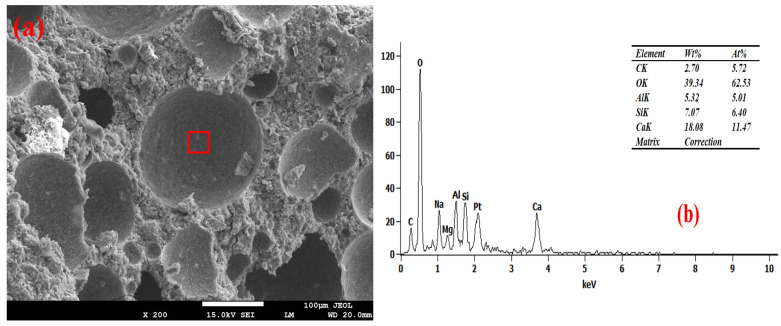
Microstructure of FC-C-G3Na. (**a**) SEM, (**b**) EDS spectrum.

**Figure 10:**
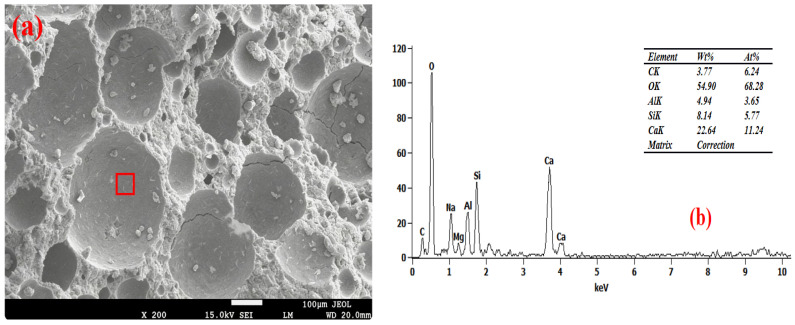
Microstructure of FC-C-G1Na. (**a**) SEM, (**b**) EDS spectrum.

Figure 7, Figure 8, Figure 9 and Figure 10 show the SEM-EDS test results of the foam concrete pore walls. The SEM-EDS results indicate that among the four groups mixed with sodium hydroxide, the pore walls of the FC-C-G7Na group exhibit a significant number of distinct needle-like structures. As the sodium hydroxide content decreases, these needle-like structures diminish noticeably. This phenomenon occurs because an increase in sodium hydroxide content drastically accelerates the dissolution rate of Si-O and Al-O bonds, leading to a substantial increase in the dissolution of aluminosilicate oligomers. At this stage, the high concentration of aluminosilicate oligomers rapidly combines with cations, causing the nucleation rate of the crystallization reaction to far exceed the crystal growth rate. This readily promotes the formation of fine and elongated needle-like crystalline phases (such as N-A-S-H, ettringite, or hydrated calcium aluminosilicate whiskers) [33,34]. Simultaneously, the elevated alkalinity increases the supersaturation of the system, making it easier for needle-like crystals to grow directionally on the gel matrix surface of the pore walls [35].

As can be seen from Figure 10, the FC-C-G1Na group exhibits numerous cracks. This is attributed to the excessively low alkali activator content, where the system’s alkalinity fails to meet the basic requirements for hydration reactions. This is manifested by insufficient hydration reactions leading to a loose and porous matrix on the pore walls [36,37], and the absence of needle-like crystalline phases results in a loss of microstructural support for the pore walls. Furthermore, the abnormally high calcium-to-silicon ratio (2.78) induces internal stress within the structure [38]. The interplay of these factors ultimately leads to the initiation and propagation of cracks on the pore walls, and these cracks further exacerbate the reduction in compressive strength for this group.

Furthermore, EDS analysis revealed that as the NaOH content decreased from 7% to 1%, the calcium-to-silicon ratio (Ca/Si) of the foamed concrete gradually increased from 2.19 to 2.78, indicating a negative correlation. Research indicates that high concentrations of alkali metal hydroxides inhibit calcium dissolution whilst promoting the leaching of amorphous silica, thereby reducing the calcium-to-silica ratio of the C-S-H gel. Consequently, at a high NaOH content (7%), the participation of calcium in the reaction is restricted, resulting in a lower Ca/Si ratio; as the NaOH content decreases, the dissolution and participation of calcium gradually recover, and the Ca/Si ratio rises accordingly [39]. A sodium hydroxide content of 3% achieved ‘moderate alkali activation’, with the calcium-to-silicon ratio falling within the optimal range of 2.56. At this point, the reaction products were predominantly high-cohesive C-(A)-S-H gels, the degree of matrix densification was optimal, and foam stability and pore structure exhibited good synergy; this may therefore be one of the reasons why the strength of this group reached its peak.

### 3.7. XRD and TG Analysis of Foam Concrete

**Figure 11:**
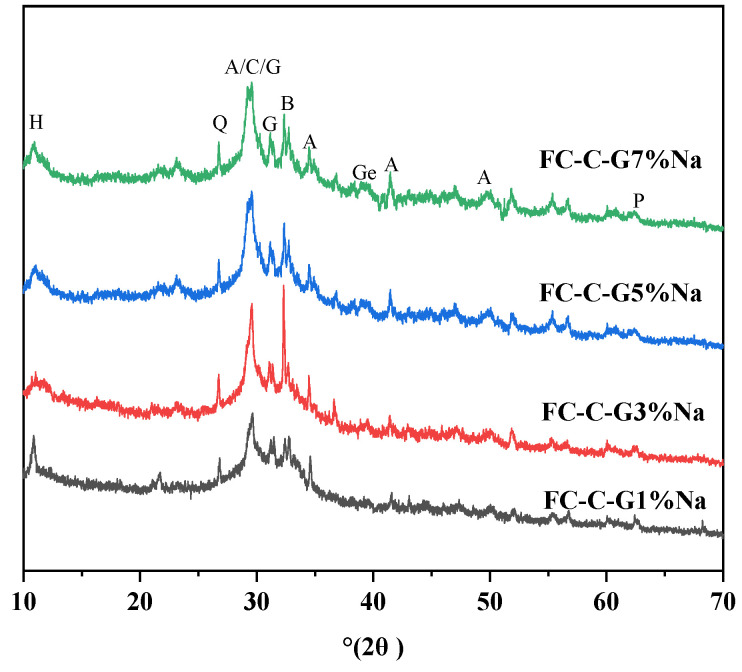
XRD Analysis of foam concrete after 3 days of curing. A: tricalcium silicate; B: anorthosilicate; C: calcite; H: hydrated calcium aluminate (Mg_6_Al_2_(OH)_16_CO_3_·4H_2_O); P: calcium hydroxide; G: hydrated calcium silicate; Q: quartz; Ge: hydrated germanium iron ore (C_2_ASH_8_).

Figure 11 shows the XRD analysis of foam concrete prepared with different activator dosages at 3 days. The diffraction peaks at 2θ values around 29° to 30° represent C-S-H and unhydrated C_3_S and C_2_S [25]. The diffraction peak at 2θ between 29° and 35° for FC-C-G3Na is sharper than those of other groups, while the FC-C-G1Na group exhibits the weakest peak intensity in this range. This indicates a significant presence of amorphous solid phases in the diffraction peak between 29° and 35° for FC-C-G1%Na. Compared to FC-C-G3%Na, the FC-C-G7%Na group exhibits slightly reduced diffraction peak intensity between 2θ values of 29° and 35°, but sharper diffraction peaks between 2θ values of 40° and 60°. This suggests that both excessive and insufficient activator content can lead to defects in the hydration products. Excessive activator accelerates the hydration reaction rate, leading to overly rapid formation of hydration products. Insufficient activator slows the reaction rate, resulting in incomplete or uneven hydration product formation. Furthermore, both excessive and insufficient activator levels compromise the stability of hydration products. Excessive activator may cause structural instability or generate undesirable reaction products in the hydration products, while insufficient activator may restrict hydration product formation, compromising its stability [40,41]. This likely explains the higher defect levels observed in the hydration products of FC-C-G1%Na and FC-C-G7%Na.

**Figure 12:**
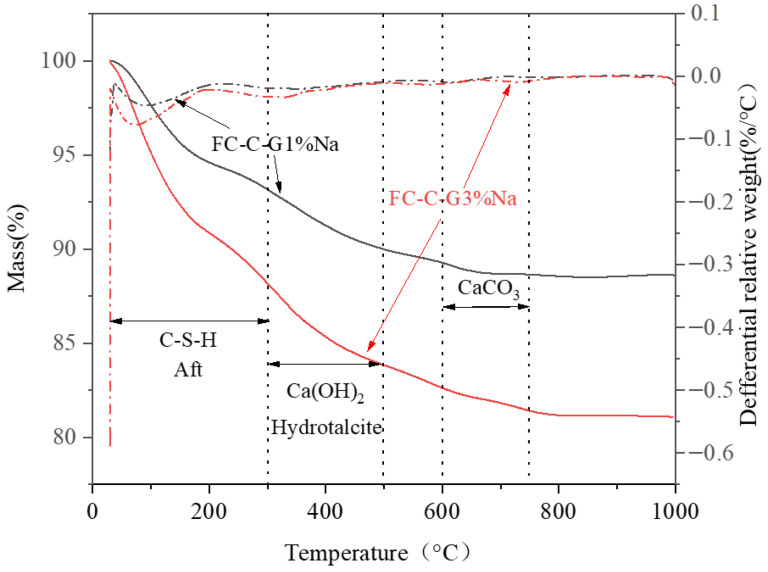
Effect of activators on quality loss in foam concrete.

Figure 12 illustrates the effect of different activator dosages on the thermogravimetric (TG/DTG) curves of foam concrete. Based on existing research [42,43], mass loss in each temperature range can be attributed as follows: the 50–300 °C range primarily corresponds to the decomposition of calcium silicate hydrate (C-S-H) and calcium aluminate hydrate (calcium aluminate hydrate); the 300–500 °C range is mainly caused by the decomposition of calcium hydroxide (Ca(OH)_2_); while the 600–750 °C range is primarily attributed to the decomposition of calcium carbonate (CaCO_3_). Compared to the FC-C-G1%Na group, the FC-C-G3%Na group exhibited a significantly higher mass loss rate in the 0–300 °C range, indicating a greater content of C-S-H gel and calcium aluminate hydrate formed. This demonstrates that a 3% sodium hydroxide content effectively penetrates into the interior of C3S and C2S particles. This process releases more Ca^2+^ and Si^4+^ ions from the precursor, leading to the dissolution of the calcium silicate phase. This dissolution accelerates the formation and precipitation of calcium hydroxide, thereby promoting the extensive formation of C-S-H gel. Ultimately, this significantly enhances the material’s early strength.

### 3.8. Compressive Strength and Water Absorption of Foam Concrete

**Figure 13:**
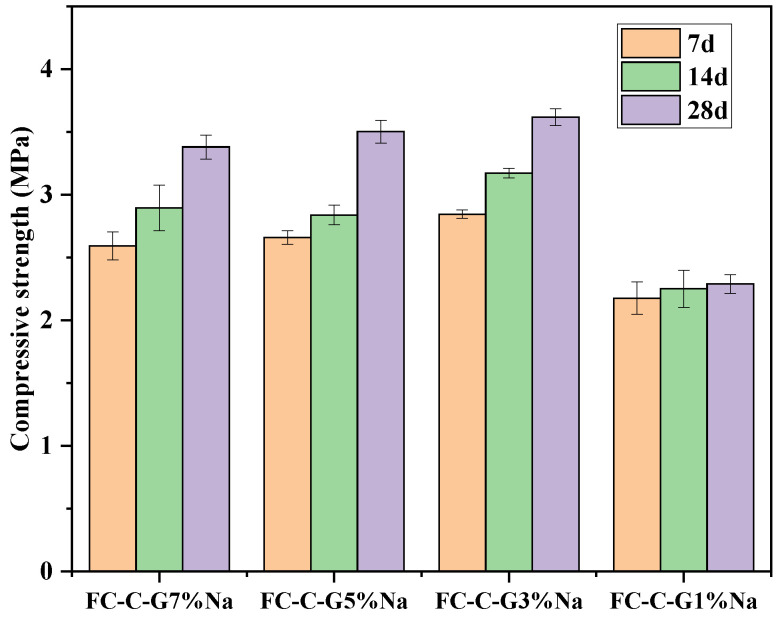
Compressive strength of foam concrete.

Figure 13 shows the compressive strength of foam concrete prepared with different activator dosages. The compressive strengths of FC-C-G7%Na, FC-C-G5%Na, FC-C-G3%Na, and FC-C-G1%Na were 3.38 MPa, 3.50 MPa, 3.62 MPa, and 2.29 MPa, respectively. Both excessive and insufficient activator dosages reduce the compressive strength of foam concrete, with FC-C-G3%Na exhibiting the highest compressive strength. The results showed that the FC-C-G3Na group exhibited the highest compressive strength at 28 days (3.62 ± 0.06 MPa) and the lowest degree of dispersion, with a 95% confidence interval of 3.56–3.68 MPa; the FC-C-G1Na group had the lowest strength (2.29 ± 0.08 MPa), with a confidence interval of 2.21–2.37 MPa; the FC-C-G5Na group (3.50 ± 0.09 MPa) and the FC-C-G7Na group (3.38 ± 0.10 MPa) had intermediate strengths, with comparable variability. One-way analysis of variance indicated that the differences in compressive strength between the groups were highly significant, confirming that the activator dosage has a genuine effect on 28-day strength ($F_{\left(3,8\right)}≅160.4,\;\rho \leq 0.001$). Post hoc comparisons suggested that the FC-C-G3Na group exhibited the highest strength, significantly higher than that of the FC-C-G1Na group; the FC-C-G5Na and FC-C-G7Na groups were intermediate, with no significant difference between them, but both were significantly higher than the FC-C-G1Na group.

**Figure 14:**
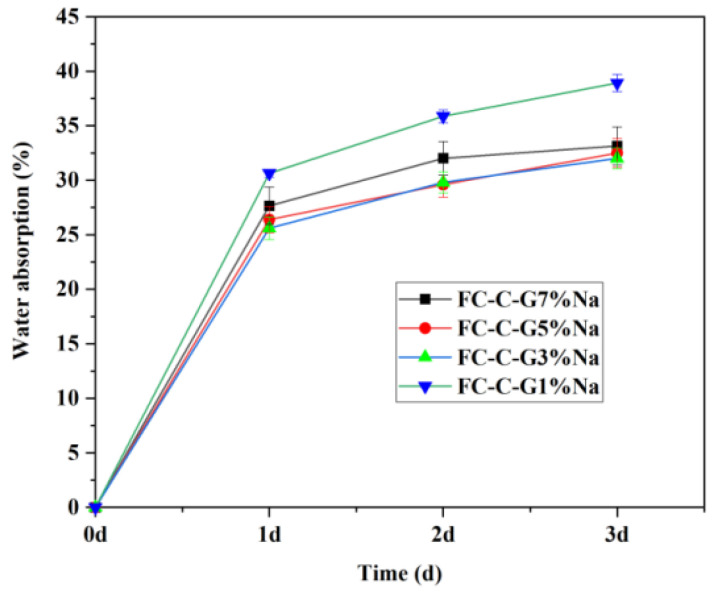
Water absorption rate of foam concrete.

Figure 14 displays the 3-day water absorption rates of foam concrete prepared with different activator dosages under composite activation methods. The 3-day water absorption rates for FC-C-G7%Na, FC-C-G5%Na, FC-C-G3%Na, and FC-C-G1%Na were 33.15%, 32.49%, 32.01%, and 38.92%, respectively. It can be observed that the FC-C-G3%Na group exhibited the lowest water absorption rate, and the higher the compressive strength of a group, the lower its water absorption rate.

This phenomenon can be reasonably explained from two aspects: the composition and structure of hydration products and the evolution of microporous structures. Both excessively high and low activator dosages significantly impact the hydration reaction process and microstructural formation, thereby determining the material’s macroscopic mechanical performance. When the activator content is too low, the alkaline environment within the system is insufficient to effectively disrupt the glass network structure in the raw materials, leading to reduced leaching rates of active SiO_2_ and Al_2_O_3_ [44,45]. Consequently, the pozzolanic reaction proceeds slowly, resulting in insufficient hydration product formation. Key gel phases like C-S-H and calcium aluminate hydrate (AFt) are produced in limited quantities, leading to poor continuity and density in the cementitious matrix. This ultimately causes a significant reduction in the compressive strength of foam concrete. Furthermore, excess alkali metal ions may precipitate as soluble salts, destabilizing hydration products and impairing C-S-H gel polymerization and spatial network formation. These factors collectively induce microstructural porosity and defect accumulation, increasing water absorption and reducing load-bearing capacity [46].

In summary, the FC-C-G3%Na group exhibits an optimal range for activator dosage. Within this range, the raw material’s reactivity is fully stimulated, promoting extensive gel formation and enhancing the compressive strength of foam concrete.

### 3.9. Drying Shrinkage

**Figure 15:**
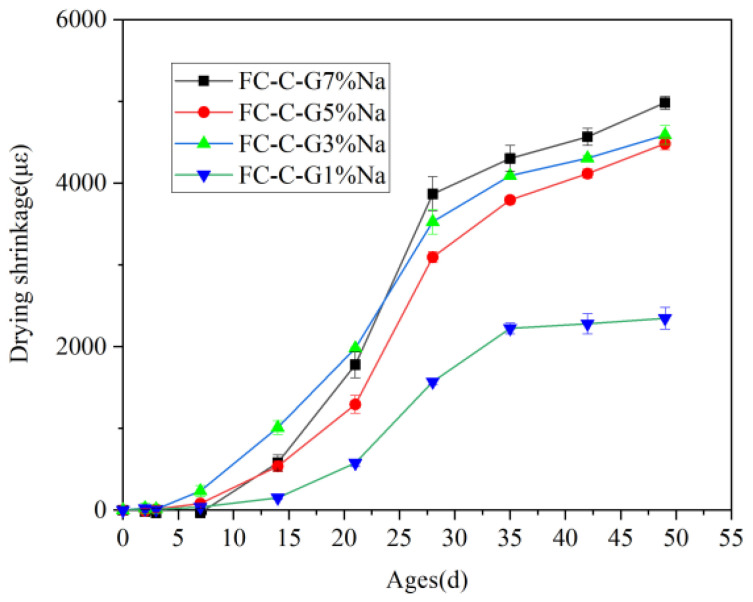
Drying shrinkage of foam concrete.

Figure 15 shows the drying shrinkage of foam concrete with different activator dosages. The 49-day drying shrinkage rates for FC-C-G7%Na, FC-C-G5%Na, FC-C-G3%Na, and FC-C-G1%Na were 4983.97 µε, 4483.28 µε, 4588.87 µε, and 2347.135 µε, respectively. Overall, drying shrinkage gradually increased with higher activator content. This occurs because higher alkali content enhances the shrinkage sensitivity of the microstructure formed through polymerization reactions (manifested as larger internal surface area, higher nanopore content, and increased ion concentration). Consequently, stronger capillary stresses and osmotic pressures act during drying, ultimately leading to greater drying shrinkage [47]. The drying shrinkage of the FC-C-G3%Na group was slightly greater than that of the FC-C-G5%Na group. This difference may stem from the former group producing the maximum amount of hydration gel. The gel matrix contains abundant nanoscale gel pores, which lose their internal adsorbed water in a drying environment, causing significant gel shrinkage.

Judging from the changes in the intensity and types of characteristic peaks in the XRD patterns shown in Figure 11, the drying shrinkage behavior of alkali-activated foamed concrete can be clearly explained: the characteristic peaks of hydration products such as A, C and G in the FC-C-G1%Na group are the weakest, indicating that the degree of hydration of the cementitious materials is extremely low at low alkali content, resulting in a low yield of gel-like products (such as C-S-H and hydrated aluminates) within the system. Consequently, the gel network of the solid-phase skeleton is underdeveloped, and the driving forces of capillary tension and chemical shrinkage are insufficient [48,49]; thus, the drying shrinkage rate after 49 days was only 2347.135 microstrain, the lowest among the four groups. The characteristic peak intensities of theFC-C-G3%Na and FC-C-G5%Na groups were moderate and similar; the formation of hydration products and the degree of crystallization were in equilibrium. The gel network formed a relatively stable spatial structure. This was due to the gel exhibiting a certain degree of capillary water absorption and shrinkage effects, whilst avoiding significant volume shrinkage caused by excessive crystallization of the products. Consequently, the drying shrinkage rates were 4588.87 and 4483.28 microstrain, which are at a moderate level and close in value. The FC-C-G7%Na group exhibited the highest characteristic peak intensity; the high alkalinity significantly accelerated the hydration process of the gel-forming material, markedly increasing both the crystallinity and yield of the hydration products. The capillary tension induced by a large amount of gel combined with the volume shrinkage of the crystalline phase, whilst lattice distortion of the products in the high-alkali environment also exacerbated the drying shrinkage. Ultimately, this resulted in a drying shrinkage rate of 4983.97 microstrain after 49 days, the highest among the four groups.

## 4. Conclusions

In this study, sodium hydroxide and sodium silicate were employed as activators to enhance the performance of foam concrete. Laser displacement sensors, XRD, TG/DTG, and optical microscopy were utilized to investigate the mechanism of action. The primary conclusions drawn from the research are:

(1) When sodium silicate and sodium hydroxide were used together as activators, increasing sodium hydroxide content reduced the development rate of matrix yield stress and decreased the stability of freshly mixed foam concrete but optimized the pore structure size.
(2) With increasing sodium hydroxide content, the compressive strength of foam concrete first increased and then decreased. Under conditions using a composite activator, adding 3% sodium hydroxide significantly increases the compressive strength of foam concrete while reducing its water absorption rate.
(3) Both excessively low and high sodium hydroxide contents disrupt the formation of an ordered gel system within the matrix, leading to poorly distributed, loosely structured hydration products. Moreover, excessive sodium hydroxide content increases drying shrinkage in foam concrete.

## Figures and Tables

**Table 1:** Composition of cement and mineral powder (wt%).

Oxide	CaO	SiO_2_	Al_2_O_3_	Fe_2_O_3_	MgO	SO_3_	K_2_O	Na_2_O	LOI
Slag	35.58	35.10	16.32	0.69	9.32	1.17	0.41	0.49	0.92
Cement	51.42	24.99	8.26	4.03	3.71	2.51	0.65	0.11	4.32

**Table 2:** Foam concrete mix proportions.

Mix	Target Density (kg/m^3^)	Cement (kg)	Slag (kg)	Water (kg)	Waterglass (kg)	Sodium Hydrate (kg)	Foam (m^3^)
FC-C-G7%Na	800	99.63	398.50	249.07	18.13	34.87	0.55
FC-C-G5%Na	800	99.63	398.50	249.07	18.13	24.91	0.55
FC-C-G3%Na	800	99.63	398.50	249.07	18.13	14.94	0.56
FC-C-G1%Na	800	99.63	398.50	249.07	18.13	4.98	0.56

**Table 3:** Matrix flow and matrix yield stress.

Mix	Spread (mm)			Yield Stress (Pa)		
	0 min	30 min	60 min	0 min	30 min	60 min
P-C-G7Na	311	292	259	4.07	5.58	10.16
P-C-G5Na	302	276	240	4.70	7.38	14.84
P-C-G3Na	285	256	234	6.27	10.72	16.80
P-C-G1Na	280	259	226	6.84	10.10	19.96

**Table 4:** Matrix setting time (min).

	FC-C-G7%Na	FC-C-G5%Na	FC-C-G3%Na	FC-C-G1%Na
Initial setting time (min)	113	135	148	157
Final setting time (min)	125	150	166	176

## Data Availability

The original contributions presented in this study are included in the article. Further inquiries can be directed to the corresponding author.
